# Correction to “Impact of Delay of Treatment With Disease‐Modifying Antirheumatic Drugs in Psoriatic Arthritis: The CorEvitas Psoriatic Arthritis/Spondyloarthritis Registry”

**DOI:** 10.1002/acr2.70104

**Published:** 2025-07-29

Mease PJ , Nowak M , Choi J , et al. Impact of delay of treatment with disease‐modifying antirheumatic drugs in psoriatic arthritis: the CorEvitas Psoriatic Arthritis/Spondyloarthritis Registry. ACR Open Rheumatol. 2025;7(6):e700019.10.1002/acr2.70019PMC1218376640548638

The graphical abstract was replaced to correct the title from “Delay of Treatment With Disease‐Modifying Antirheumatic Drugs in Psoriatic Arthritis: The CorEvitas Psoriatic Arthritis/Spondyloarthritis Registry” to “Impact of Delay of Treatment With Disease‐Modifying Antirheumatic Drugs in Psoriatic Arthritis: The CorEvitas Psoriatic Arthritis/Spondyloarthritis Registry”

In the ADDITIONAL DISCLOSURES section, author Jiyoon Choi was moved from the sentence disclosing “employee of Bristol Myers Squibb” to “employee of Bristol Myers Squibb at the time of study conduct.”

We apologize for these errors.

